# Multilocus phylogeny of the parasitic wasps in the tribe Euphorini (Hymenoptera: Braconidae) with revised generic classifications

**DOI:** 10.7717/peerj.4783

**Published:** 2018-05-21

**Authors:** Yuanmeng Miles Zhang, Julia Stigenberg, Jacqueline Hope Meyer, Barbara Jo-Anne Sharanowski

**Affiliations:** 1 Department of Biology, University of Central Florida, Orlando, FL, United States of America; 2 Department of Zoology, Swedish Museum of Natural History, Stockholm, Sweden

**Keywords:** *Leiophron*, *Mama*, *Peristenus*, Taxonomy, Euphorinae, Phylogenetics, Parasitoid, *Euphoriella*, *Euphorus*, *Euphoriana*

## Abstract

**Background:**

Parasitic wasps in the family Braconidae are important regulators of insect pests, particularly in forest and agroecosystems. Within Braconidae, wasps in the tribe Euphorini (Euphorinae) attack economically damaging plant bugs (Miridae) that are major pests of field and vegetable crops. However, the evolutionary relationships of this tribe have been historically problematic. Most generic concepts have been based on ambiguous morphological characters which often leads to misidentification, complicating their use in biological control.

**Methods:**

Using a combination of three genes (*COI*, *28S*, and *CAD*) and 80 taxa collected worldwide, we conducted Bayesian inference using MrBayes, and maximum likelihood analyses using RAxML and IQ-Tree on individual gene trees as well as the concatenated dataset.

**Results:**

The monophyly of the tribe Euphorini and the two genera *Peristenus* and *Leiophron* were confirmed using maximum likelihood and Bayesian inference. The subgeneric classifications of *Leiophron sensu lato* were not supported, and the monotypic genus *Mama* was also not supported.

**Discussion:**

*Euphoriella*,* Euphoriana*, *Euphorus*, and *Mama*** syn. n,** have been synonymized under *Leiophron. Mama mariae* **syn. n** was placed as a junior synonym of *Leiophron reclinator*. The generic concepts of *Peristenus* and *Leiophron* were refined to reflect the updated phylogeny. Further we discuss the need for revising Euphorini given the number of undescribed species within the tribe.

## Introduction

Braconid wasps in the large and diverse subfamily Euphorinae is divided into 14 tribes and 52 genera (Stigenberg, Boring & Ronquist, 2015). Euphorines attack a variety of host life stages ranging from nymphal/larval hosts to adults of seven different orders of insects: Coleoptera, Hemiptera, Hymenoptera, Neuroptera, Orthoptera, Psocodea, and Lepidoptera (Shaw, 1988; Chen & Van Achterberg, 1997; Stigenberg, Boring & Ronquist, 2015). The tribe Euphorini Foerster contains koinobiont endoparasitoids of Hemiptera and Psocodea, which attack young nymphs (1st or 2nd instar) and feed internally on the hemolymph of their hosts (Loan, 1974b). Mature parasitoid larvae emerge from mature host nymphs or teneral adults, and overwinter as pupae in soil (Loan, 1974b). Several species of Euphorini have been extensively studied for their use in biological control programs because they attack many serious agricultural pests such as *Lygus* Hahn (Day, 1987; Haye et al., 2005; Haye et al., 2007). Despite the research interest using Euphorini wasps in applied entomology, the classification and identification of these parasitoids remains challenging. Variable morphological characters such as the degree of completion of the occipital carina, and the presence or absence of vein (RS + M)a and cu-a were used as defining characters (Chen & Van Achterberg, 1997; Stigenberg, Boring & Ronquist, 2015), leading to taxonomic uncertainty and misidentification.

The taxonomic history of Euphorini is a long and convoluted one. *Euphoriana* Gahan, *Euphoriella* Ashmead, *Euphorus* Nees, and *Peristenus* Foerster have all been synonymized under or treated as subgenera of *Leiophron* in a variety of combinations by different authors (Marshall, 1887; Muesebeck, 1936; Loan, 1974a; Shaw, 1985; Shaw, 1987; Chen & Van Achterberg, 1997; Stigenberg, Boring & Ronquist, 2015). In addition, *Aridelus* Marshall, *Chrysopophthorus* Goidanich, *Cryptoxilos* Viereck, *Holdawayella* Loan, *Mama* Belokobylskij, and *Wesmaelia* Foerster were also included in Euphorini until the most recent comprehensive revision of the entire subfamily Euphorinae by Stigenberg, Boring & Ronquist (2015). Only a few exemplars of Euphorini were included in the study, but the monophyly of the tribe was strongly supported. Currently there are three recognized genera within Euphorini, including *Leiophron sensu lato*, *Peristenus*, and the monotypic *Mama* (Stigenberg, Boring & Ronquist, 2015). *Leiophron* is further divided into four subgenera, *Euphoriana*, *Euphoriella*, *Euphorus*, and *Leiophron sensu stricto* (Stigenberg, Boring & Ronquist, 2015).

Here, we extensively sample Euphorini wasps and reconstruct the evolutionary relationships among its members using a multi-locus dataset. We reassess the generic and subgeneric concepts of Euphorini and revise the classification to reflect the phylogeny. The results of this study provide a comprehensive framework for phylogenetic relationships among Euphorini wasps and we provide taxonomic clarity and identification resources to aid future applied research and biological control programs.

## Materials & Methods

### Sample collection

**Figure 1:**
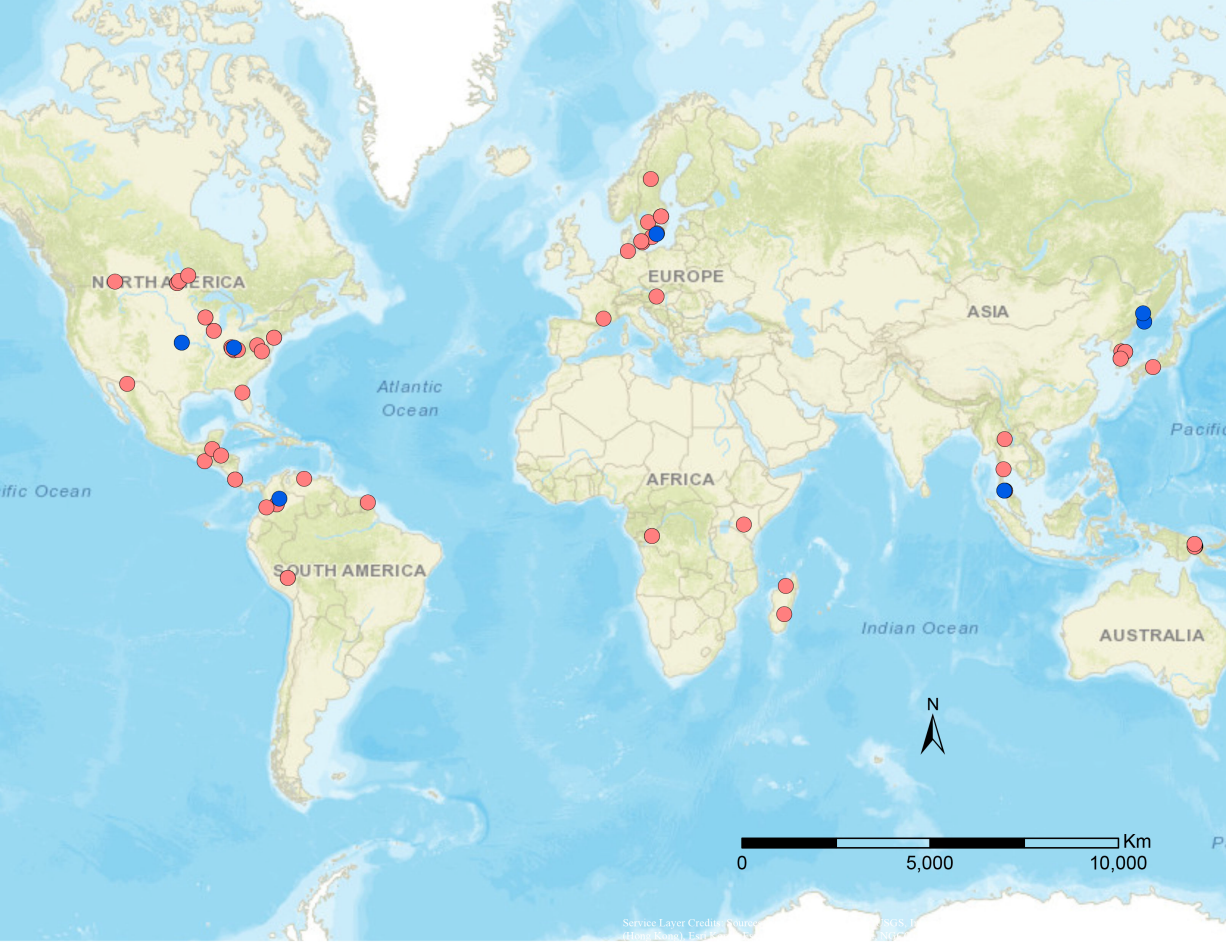
Geographical distribution of specimens used in this study. Blue dots are published data from Stigenberg, Boring & Ronquist (2015), red dots are newly sampled taxa for this study.

Specimens were borrowed from the following institutions and curators: Hymenoptera Institute Collection, University of Kentucky (HIC, M. Sharkey), French National Museum of Natural History (MNHN, C. Villemant), Swedish Museum of Natural History (NHRS, H. Vårdal), and Zoological Institute of Russian Academy of Sciences (ZIN, S. Belokobylskij). Additional specimens were collected via sweep netting or Malaise trap samples from Canada, USA, and Peru. Specimens were identified using Chen & Van Achterberg (1997), Loan (1974a), Loan (1974b), Stigenberg & Van Achterberg (2016). Outgroups included representatives of the most closely related tribes to Euphorini, based on Stigenberg, Boring & Ronquist (2015): *Microctonus* Wesmael (Perilitini), *Townesilitus* Haeselbarth & Loan (Townsilitini), and *Chrysopophthorus* Goidanich (Helorimorphini). A list of the specimens utilized in this study is provided in Table 1, and detailed locality information in Table S1. A map of the distribution of these specimens is depicted in Fig. 1, which was generated using ArcMap v10.5.1. For ease of interpretation of results, specimen information was added to taxon labels for the phylogenetic analyses, including country and lowest identification. The subgeneric names within *Leiophron s.l.* are used as specimen names to avoid confusion with *Leiophron s.s.* (eg. *Leiophron (Leiophron) uniformis* is listed as *Leiophron uniformis*, whereas *Leiophron (Euphoriana) dispar* is listed as *Euphoriana dispar*).

**Table 1:** Specimen information. GenBank accession numbers (new sequences generated for this study is in bold), collection localities, and voucher deposition institutes for all specimens used in this study. HIC (Hymenoptera Institute, University of Kentucky, Lexington), MNHN (French National Museum of Natural History, Paris), NHRS (Swedish Museum of Natural History, Stockholm), UCFC (University of Central Florida Collection of Arthropods, Orlando), ZIN (Zoological Institute of Russian Academy of Sciences, St. Petersburg).

**Taxon Label**	***COI***	***28S***	***CAD***	**Locality**	**Voucher location**
07_Yves_Leiophron_PNG	MG926854	MG913702	–	Papua New Guinea	MNHN
08_Yves_Leiophron_PNG	MG926855	MG913703	–	Papua New Guinea	MNHN
10_Yves_Leiophron_PNG	MG926856	MG913704	–	Papua New Guinea	MNHN
AB016_Peristenus_KS	KJ591487	KJ591282	–	USA	HIC
AB020_Peristenus_KY	KJ591488	KJ591283	–	USA	HIC
AB023_Peristenus_KS	KJ591489	KJ591284	–	USA	HIC
Euph_001_Euphorus_pallidistigma_SWE	MG926857	–	MG913762	Sweden	NHRS
Euph_017_Peristenus_JAP	MG926858	–	MG913763	Japan	NHRS
Euph_020_Peristenus_SWE	MG926859	MG913705	MG913764	Sweden	NHRS
Euph_083_Peristenus_HUN	MG926860	MG913706	–	Hungary	NHRS
Euph_162_Leiophron_apicalis_SWE	MG926861	–	–	Sweden	NHRS
JS01000238_Leiophron_fascipennis_SWE	MG926862	MG913713	–	Sweden	NHRS
JS01000242_Leiophron_SWE	KJ591452	KJ591243	–	Sweden	NHRS
JS01000267_Leiophron_FRGU	MG926863	MG913707	–	French Guiana	NHRS
JS01000499_Mama_mariae_RUS	KJ591460	KJ591250	–	Russia	ZIN
JS01000515_Euphoriana_dispar_RUS	KJ591458	MG913708	–	Russia	ZIN
JS01000538_Euphorus_duploclaviventris_SWE	MG926864	–	–	Sweden	NHRS
JS01000539_Euphorus_oblitus_SWE	MG926865	–	–	Sweden	NHRS
JS01000540_Leiophron_deficiens_SWE	MG926866	MG913709	–	Sweden	NHRS
JS01000542_Leiophron_reclinator_SWE	MG926867	–	–	Sweden	NHRS
JS01000547_ Leiophron _MAD	MG926868	MG913710	–	Madagascar	NHRS
JS01000552_Peristenus_SWE	MG926869	MG913711	MG913765	Sweden	NHRS
JS01000553_Euphorus_basalis_SWE	MG926870	–	MG913766	Sweden	NHRS
JS01000554_Euphorus_fulvipes_SWE	MG926871	MG913712	MG913767	Sweden	NHRS
JS068_Leiophron_COL	KJ591455	KJ591246	KJ591362	Colombia	HIC
JS120_Leiophron_THA	KJ591456	KJ591247	KJ591363	Thailand	HIC
JS129_Leiophron_THA	KJ591457	KJ591248	KJ591364	Thailand	HIC
PNG_5_Leiophron	MG926872	MG913714	–	Papua New Guinea	MNHN
PNG_6_Leiophron	MG926873	MG913715	–	Papua New Guinea	MNHN
PNG_7_Leiophron	MG926874	MG913716	–	Papua New Guinea	MNHN
YMZ038_Peristenus_GER	MG926875	MG913717	MG913768	Germany	UCFC
YMZ077_Leiophron_uniformis_MB	–	MG913718	MG913769	Canada	UCFC
YMZ081_Euphoriella_MB	MG926876	MG913719	MG913770	Canada	UCFC
YMZ124_Peristenus_mellipes_MB	KY566090	MG913720	MG913771	Canada	UCFC
YMZ132_Leiophron_KY	MG926877	MG913721	MG913772	USA	UCFC
YMZ133_Leiophron_KY	MG926878	MG913722	MG913773	USA	UCFC
YMZ134_Leiophron_WV	MG926879	MG913723	MG913774	USA	UCFC
YMZ136_Leiophron_KY	MG926880	MG913724	MG913775	USA	UCFC
YMZ139_Leiophron_uniformis_FRA	MG926881	MG913725	–	France	UCFC
YMZ141_Leiophron_THA	MG926882	MG913726	MG913776	Thailand	UCFC
YMZ142_Peristenus_MAD	MG926883	MG913727	–	Madagascar	UCFC
YMZ145_Leiophron_COL	MG926884	MG913728	MG913777	Colombia	UCFC
YMZ146_Leiophron_COL	MG926885	MG913729	–	Colombia	UCFC
YMZ148_Euphoriella_GUA	MG926886	MG913730	MG913778	Guatemala	UCFC
YMZ211_Peristenus_dayi_MB	KY566098	MG913731	MG913779	Canada	UCFC
YMZ335_Peristenus_howardi_AB	KY566100	MG913732	MG913780	Canada	UCFC
YMZ341_Peristenus_relictus	KY566106	MG913733	MG913781	USA	UCFC
YMZ343_Peristenus_digoneuti	MG926887	MG913734	MG913782	USA	UCFC
YMZ345_Leiophron_KY	MG926888	MG913735	–	USA	UCFC
YMZ346_Peristenus_WI	MG926889	MG913736	–	USA	UCFC
YMZ349_Peristenus_IL	MG926890	MG913738	–	USA	UCFC
YMZ351_Leiophron_VA	MG926891	MG913739	MG913783	USA	UCFC
YMZ356_Peristenus_WI	MG926892	MG913740	–	USA	UCFC
YMZ358_Euphoriella_KY	MG926893	MG913741	–	USA	UCFC
YMZ359_Euphoriella_FL	MG926894	MG913742	–	USA	UCFC
YMZ361_Leiophron_AZ	MG926895	MG913743	–	USA	UCFC
YMZ363_Euphoriella_COL	MG926896	MG913744	MG913784	Colombia	UCFC
YMZ364_ Leiophron _CR	MG926897	MG913745	–	Costa Rica	UCFC
YMZ365_Euphoriella_CR	MG926898	MG913746	–	Costa Rica	UCFC
YMZ366_Euphoriella_GUA	MG926899	MG913747	MG913785	Guatemala	UCFC
YMZ367_Leiophron_HON	MG926900	MG913748	–	Honduras	UCFC
YMZ368_Leiophron_VEN	MG926901	MG913749	–	Venezuela	UCFC
YMZ370_Euphoriella_PER	MG926902	MG913750	–	Peru	UCFC
YMZ371_Leiophron_PER	MG926903	MG913751	MG913786	Peru	UCFC
YMZ372_Peristenus_PER	MG926904	MG913752	–	Peru	UCFC
YMZ373_Leiophron_THA	MG926905	–	MG913787	Thailand	UCFC
YMZ375_Leiophron_KEN	MG926906	–		Kenya	UCFC
YMZ376_Leiophron_THA	MG926907	MG914753	–	Thailand	UCFC
YMZ377_Leiophron_THA	MG926908	MG914754	MG913788	Thailand	UCFC
YMZ378_Leiophron_THA	MG926909	MG914755	MG913789	Thailand	UCFC
YMZ380_Leiophron_CON	MG926910	MG914756	MG913790	Congo	UCFC
YMZ382_Leiophron_KOR	MG926911	–	MG913791	South Korea	UCFC
YMZ383_Leiophron_KOR	MG926912	–	MG913792	South Korea	UCFC
YMZ384_Leiophron_KOR	MG926913	MG914757	–	South Korea	UCFC
YMZ385_Leiophron_KOR	MG926914	MG914758	–	South Korea	UCFC
YMZ386_Leiophron_CON	MG926915	MG914759	–	Congo	UCFC
YMZ388_Leiophron_pallidistigma_KOR	MG926916	MG914760	–	South Korea	UCFC
AB102 Microctonus (Perilitini)	KJ591529	KJ591329	KJ591412	USA	HIC
JS01000218 Townesilitus (Townesilitini)	KJ591440	KJ591228	KJ591353	Sweden	HIC
JS115 Chrysopophthorus (Helorimorphini)	MG926854	MG913702	–	Colombia	HIC

### Terminology and image capture

Terminology used for most morphological characters follows Chen & Van Achterberg (1997) and Stigenberg, Boring & Ronquist (2015). However, wing venation terminology follows Sharkey & Wharton (1997). Specimens were photographed using a Canon 7D Mark II with a Mitutoyo M Plan Apo 10× objective mounted onto the Canon EF Telephoto 70–200 mm zoom lens, and the Canon MT–24EX Macro Twin Lite Flash (Tokyo, Japan) with custom-made diffusers to minimize hot spots.

### DNA protocols

A total of 80 taxa were sampled out of which three species represented outgroups. The taxon sampling covers the entire range of Euphorini, which is found on all continents except in Antarctica and Australia outside of Papua New Guinea (Yu, Van Achterberg & Horstmann, 2012). This is the largest sampling of any of the euphorine tribes, and is comprised of all three Euphorini genera, as well as all four subgenera of *Leiophron s.l.* Specimens were extracted, amplified, and sequenced either at the Molecular Systematics Laboratory, Swedish Museum of Natural History following the protocols listed in (Stigenberg, Boring & Ronquist, 2015), or at the Insect Systematics Laboratory at the University of Central Florida following the DNeasy™ Tissue Kit protocol (Qiagen, Valencia, CA, USA). Petioles were separated from mesosomas to ensure buffer penetration during tissue lysis, and the two body parts were mounted onto the same point post-extraction for vouchering. Voucher specimens deposition are listed in Table 1. Three genes were amplified: partial *28S* domain 2 and 3 (rDNA), partial *CAD* (Carbamoyl-Phosphate Synthetase 2, Aspartate Transcarbamylase, and Dihydroorotase) and the 5′ region of mitochondrial *COI*. New Euphorini-specific primers were designed for *CAD* based on sequences from Sharanowski, Dowling & Sharkey (2011) and Stigenberg, Boring & Ronquist (2015). The faster rate of evolution of the mitochondrial genes is ideal for separating closely related species (Zhang, Ridenbaugh & Sharanowski, 2017), while the ribosomal and nuclear genes have slower rates of evolution and are more suitable for higher level phylogenetic relationships (Sharanowski, Dowling & Sharkey, 2011). All three genes are commonly used in Braconidae phylogenetics, including Euphorinae (Sharanowski, Dowling & Sharkey, 2011; Stigenberg, Boring & Ronquist, 2015; Zhang, Ridenbaugh & Sharanowski, 2017).

All PCRs were performed on a Bio-Rad MyCyclerTM thermal cycler, using approximately 1µg DNA extract, 1 × Standard Taq Buffer (10 mm Tris-HCl, 50 mm KCl, 1.5 mm MgCl_2_, pH 8.3, New England Biolabs, Ipswich, Massachusetts, USA), 200 *μ*M dNTP (Invitrogen, Carlsbad, CA, USA), 4 mM MgSO_4_, 400 nM of each primer, 1 unit of Taq DNA polymerase (New England Biolabs), and purified water to a final volume of 25 µl. Primer information and PCR conditions are listed in Table 2. Amplicons of reaction products were cleaned with Agencourt CleanSEQ magnetic beads and sequenced in both directions using the BigDye Terminator Cycle Sequencing Kit (Applied Biosystems, USA) and the Applied Biosystems 3730xl DNA Analyzer at the University of Kentucky, Advanced Genetic Technologies Center (UK-AGTC). Contigs were assembled and edited using Geneious version 8.18 (Kearse et al., 2012), and alignment was conducted using MAFFT server (Katoh et al., 2002) (https://mafft.cbrc.jp/alignment/server/). The protein coding genes were aligned using default MAFFT settings, and for *28S* we used Q-INS-I strategy (Katoh & Toh, 2008) which takes secondary RNA structure into account. New sequences obtained from this study were deposited in GenBank (See Table 1).

**Table 2:** Primers. List of primers used in this study.

**Gene region**	**Primers**	**Sequence (5′ to 3′)**	**Source**	**Annealing temperature**
*28S*	D2F (fwd)	AGTCGTGTTGCTTGATAGTGCAG	Campbell, Steffen-Campbell & Werren (1994)	55 °C
	D2R (rev)	TTGGTCCGTGTTTCAAGACGGG	Campbell, Steffen-Campbell & Werren (1994)	55 °C
*COI*	LCO1490 (fwd)	GGTCAACAAATCATAAAGATATTGG	Folmer et al. (1994)	49 °C
	HCO2198 (rev)	TAAACTTCAGGGTGACCAAAAAATCA	Folmer et al. (1994)	49 °C
*CAD*	CAD2F (fwd)	TAYGAGCTTACCAAAATWGAYC	New primer	52 °C
	CAD2R (rev)	CATAAATGTCCATCACAACTTC	New primer	52 °C

### Phylogenetic analyses

The three genes were analyzed separately and concatenated using Bayesian inference (BI) analysis with MrBayes v3.2.6 (Ronquist et al., 2012) on the CIPRES Science Gateway (Miller et al., 2009). Each analysis had two independent searches with four chains and were run for 10,000,000 generations, sampling every 1,000, with a 10% burnin discarded. For the concatenated analysis, partitions were separated by gene and codon position for protein-coding genes for a total of six preselected partitions. Six partitions (*28S*; *CAD_1*; *CAD_2 +CAD_3*; *COI_1*; *COI_2*; and *COI_3*) were chosen using PartitionFinder 2.1.1 (Lanfear et al., 2016), based on the greedy algorithm and Bayesian Information Criterion (Table 3). The same partitions were also used for a maximum likelihood (ML) analysis using the relatively new IQ-Tree method (Nguyen et al., 2014), with 1,000 ultrafast bootstraps developed by Hoang et al. (2018). The concatenated dataset was also analyzed with RAxML v8.2.0 (Stamatakis, 2006), using the GTR+ Γ model of nucleotide substitution and 1,000 nonparametric bootstraps. All alignment files and scripts files can be accessed on Figshare (https://figshare.com/articles/Concatenated_File/6160247). All resulting trees were visualized using FigTree v1.4.2 (Rambaut, 2012). Intraspecific distances between *Mama mariae* Belokobylskij and *Leiophron reclinator* (Ruthe) was calculated using MEGA v7.0.21 (Kumar, Stecher & Tamura, 2016) using the Kimura-2-parameter (K2P) model (Kimura, 1980).

## Results

**Figure 2:**
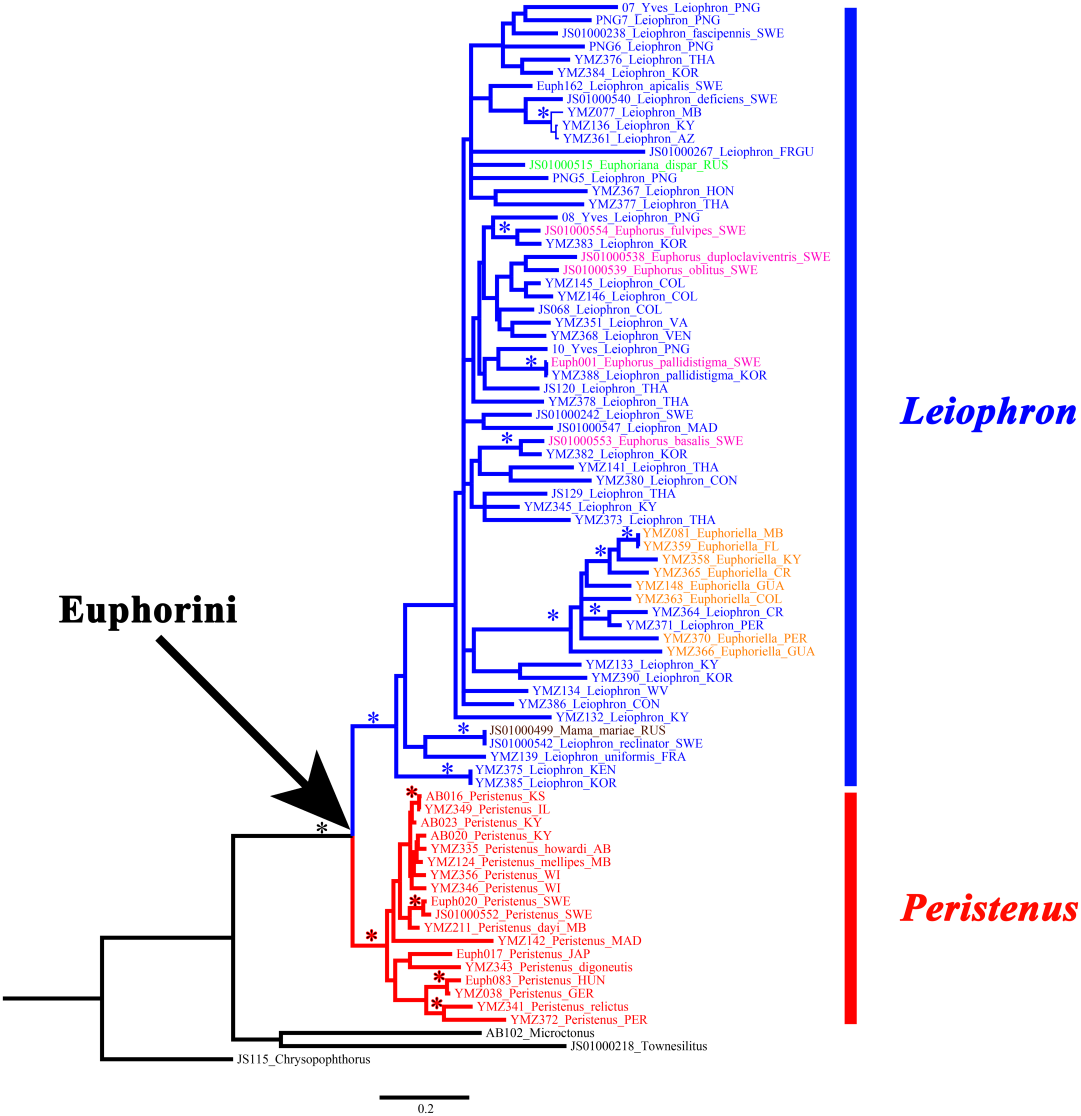
Concatenated gene tree for MrBayes, RAxML, and IQ-Tree. *Peristenus* is colored red, and *Leiophron* is colored in blue, with subgenera within *Leiophron* shown in different colors (*Leiophron sensu stricto* in blue, *Euphorus* in purple, *Euphoriana* in green, *Euphoriella* in orange, and *Mama* in brown). Asterisks indicate strong nodal support for all three analyses (≥0.98 posterior probability support for MrBayes; ≥90 for bootstrap support for RAxML; and ≥90 for ultrafast bootstrap support for IQ-Tree).

Here we present the most taxonomically comprehensive phylogeny of the euphorine braconid tribe Euphorini with all known genera and subgenera sampled. All genera and subgenera had multiple representatives except *Euphoriana* (only one exemplar included - *E*. *dispar*) and the monotypic *Mama mariae*. A total of 39 *CAD*, 71 *28S*, and 80 *COI* for a total of 190 sequences were used for the final analyses, 158 of which were newly generated for this study (Table 1). The summary statistics of all three genes can be found in Table 3. While we failed to amplify *CAD* sequences from some older specimens, the gene itself is informative (See Table 3) and should be used in other multilocus analyses of braconids. All the individual BI gene trees (Figs. S1–S3), as well as the concatenated BI and ML (Figs. S4–S6) analyses strongly supported the monophyly of the tribe Euphorini (1, 100, 100, for MrBayes posterior probability, RAxML bootstrap support, and IQ-Tree ultrafast bootstrap support, respectively) as well as the monophyly of the genera *Peristenus* (1, 95, 99) and *Leiophron s.l.* (1, 90, 90) (Fig. 2)*.*

**Table 3:** Marker information. Markers, partitions schemes, and substitution models (Model) according to PartitionFinder2. Additional summary includes number of basepairs (#bp), number of variable sites (#var), number of parsimonious informative sites (#par), and the CG content (CG%).

**Marker and partitions**	**#bp**	**#var**	**#par**	**CG%**	**Model**	**References**
**28S nuclear DNA**						
28S_123	592	263	179	43.3	GTR+G	Tavaré (1986)
**CAD nuclear DNA**	507	185	121	36.0		
CAD_1	169	133	96	26.5	HKY+I+G	Hasegawa, Kishino & Yano (1985)
CAD_23	338	52	25	40.3	HKY+I+G	Hasegawa, Kishino & Yano (1985)
**COI mtDNA**	660	360	296	28.4		
COI_1	220	103	78	33.8	GTR+I+G	Tavaré (1986)
COI_2	220	55	33	38.4	GTR+G	Tavaré (1986)
COI_3	220	202	185	12.8	GTR+I+G	Tavaré (1986)

*Mama mariae*, the only species from the monotypic genus *Mama*, was not supported as a distinct genus and was instead recovered as a sister group of *Leiophron reclinator* (Fig. 2) with 0.8% divergence among *COI* intraspecific genetic distance.

The four subgenera of *Leiophron s.l.* (*Leiophron s.s.*, *Euphoriana*, *Euphoriella*, *Euphorus*) were not supported as distinct clades within the monophyletic *Leiophron s.l.* in any of the phylogenetic analyses (Fig. 2, Figs. S1–S5).

## Discussion

### Generic concepts of *Peristenus* and *Leiophron s.l.*

**Figure 3:**
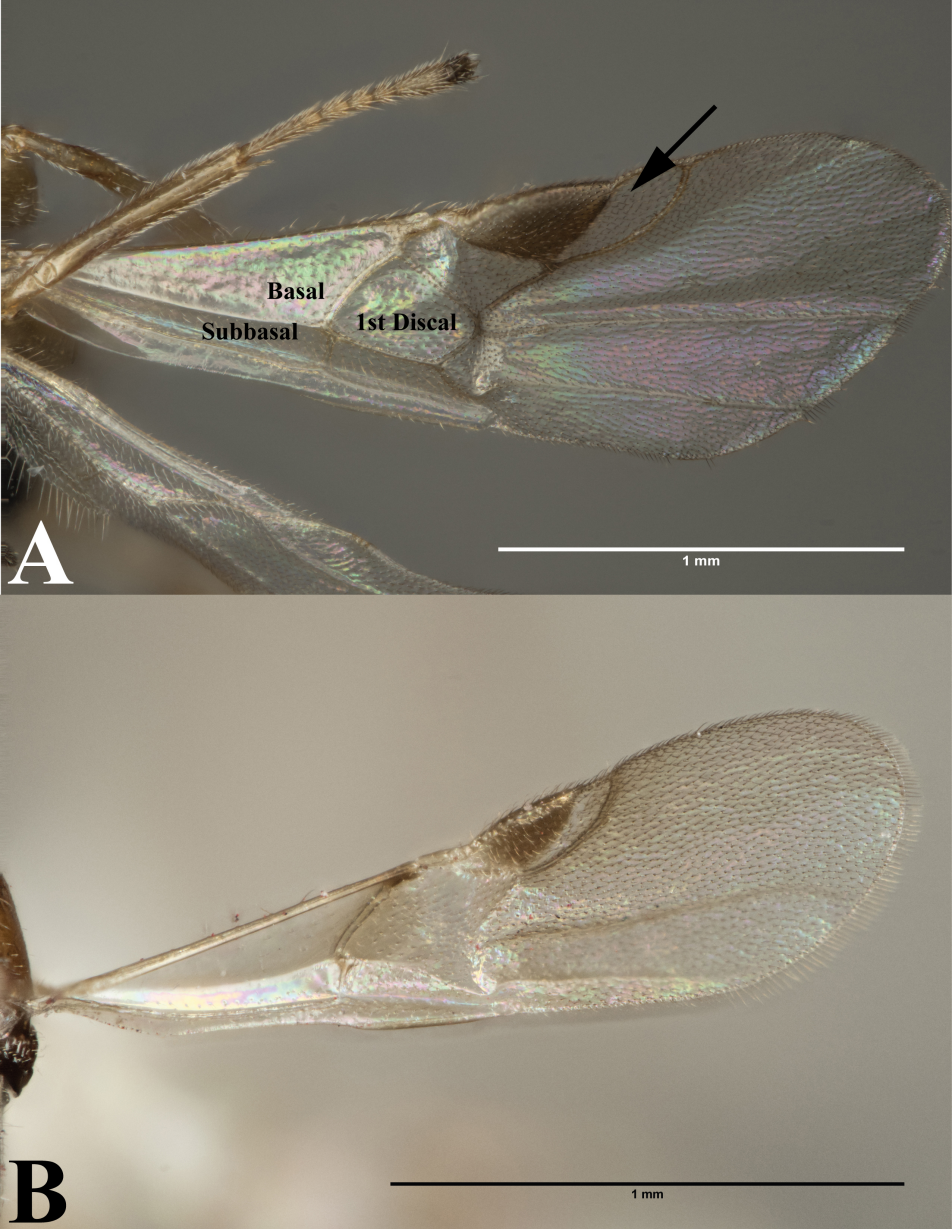
Forewing. (A) Forewing of *Peristenus*; arrowing pointing to marginal cell. (B) Forewing of *Leiophron*. Photos by JH Meyer.

**Figure 4:**
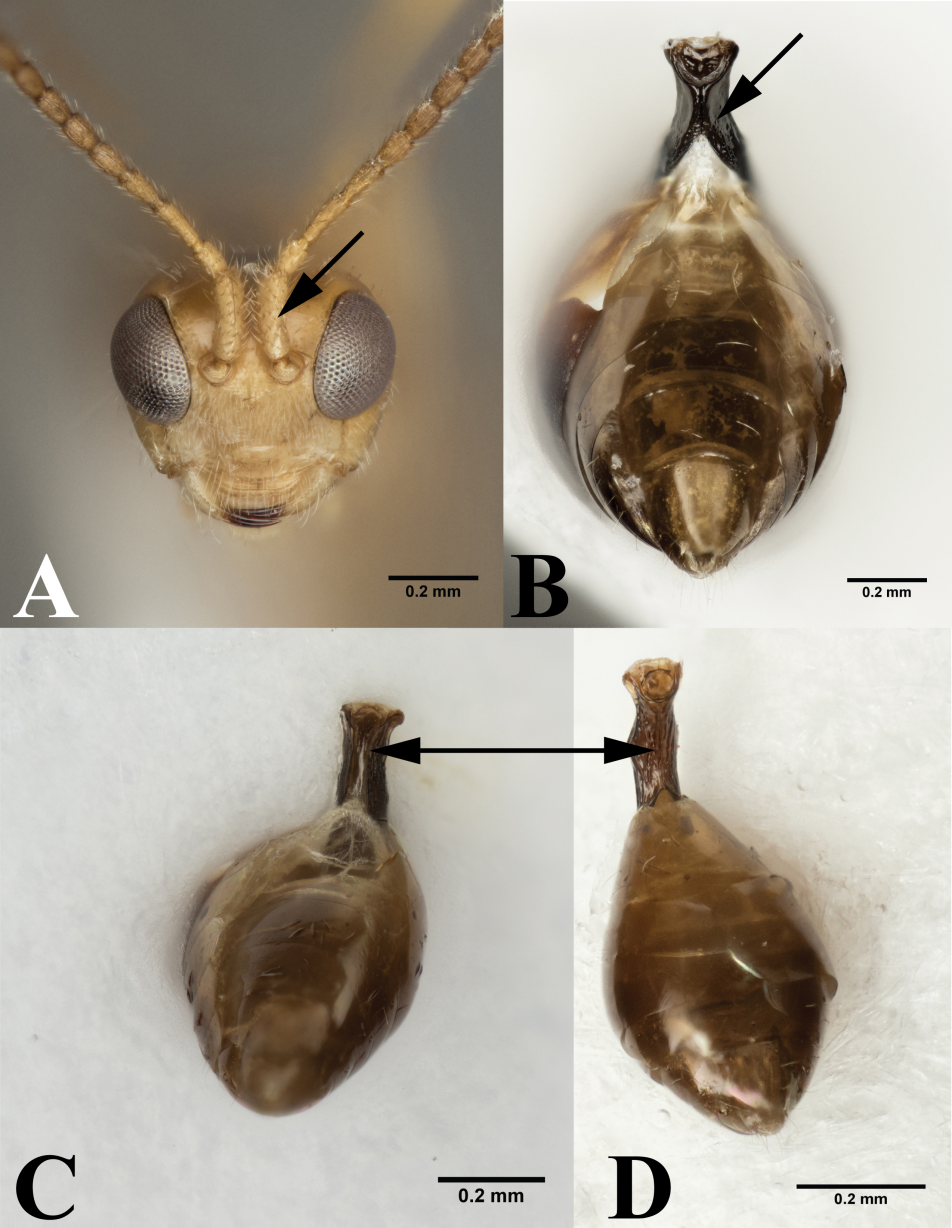
*Leiophron* vs *Peristenus*. (A) Frontal view of *Leiophron reclinator* (*Mama mariae* **syn. n)**, arrow pointing to spiny scape of antennae. (B) Ventral view of* Peristenus* metasoma, arrow pointing to the partially fused petiole. Ventral view of *Leiophron* metasoma: (C) arrowing pointing to ventral petiole showing unfused sclerite at midline. (D) arrowing pointing to completely fused sclerite at midline of petiole. Photos by JH Meyer.

Our data supports the monophyly of *Peristenus* and *Leiophron*, corroborating the results of previous morphological studies (Loan, 1974a; Loan, 1974b; Shaw, 1985; Shaw, 1987; Chen & Van Achterberg, 1997). Additionally, with a much more focused taxon sampling we were able to delineate the finer relationships within the tribe Euphorini. *Peristenus* is largely uniform in morphology and exclusively attacks Miridae, while its sister taxon *Leiophron* is much more variable in both morphology and host breadth which likely has led to convergent morphology, and hence the subgeneric concepts and taxonomic confusion. *Peristenus* can be distinguished from *Leiophron* by the evenly setose 1st discal, basal, and subbasal cells in the forewing (Fig. 3A), and the 1st metasomal tergite, which is fused or touching basally (Fig. 4B).

Representatives of all four subgenera of *Leiophron s.l.* defined by Stigenberg, Boring & Ronquist (2015): *Euphoriana*, *Euphoriella*, *Euphorus*, and *Leiophron s.s.*, were included in this analysis. These subgeneric relationships were not supported in any of our analyses, as they failed to form monophyletic clades (Fig. 2). This is not surprising given the lack of consistent morphological characters as specimens often exhibit characteristics of different subgenera, leading to similar suggestions for synonymizations in the past (Muesebeck, 1936; Loan, 1974b; Shaw, 1985; Stigenberg, Boring & Ronquist, 2015). Morphological characters such as the presence or absence of the forewing vein (RS + M)a, hindwing vein cu-a, and complete occipital carina are all too variable to be used as defining characteristics (Chen & Van Achterberg, 1997; Stigenberg, Boring & Ronquist, 2015). These ambiguous distinguishing characters at the subgeneric level can easily lead to misidentification in ecological or applied studies. *Euphoriella* is morphologically unique based on the absence of radial cell, and the fusion of ventral side of petiole. However, this subgenus is rendered paraphyletic by *Leiophron* sp. YMZ364 and YMZ371. The type species of *Euphoriella*, *Euphoriella incerta* Ashmead, was originally collected from Florida (Muesebeck, 1936). However, the type specimen is too badly damaged to be compared with our equally damaged DNA voucher also collected from Florida to accurately confirm its identity. While there are no nomenclatural rules that requires subgenera to be monophyletic, we do not support paraphyly as these morphological similarities could be the result of convergent evolution and does not reflect evolutionary history. Therefore, based on our molecular evidence combined with the inconsistency of previously used morphological characters, we recommend treating *Leiophron* as a single genus without further subdivisions and synonymize the subgenera *Euphoriana*, *Euphoriella*, and *Euphorus* as junior synonyms of *Leiophron*. With this taxonomic update, *Leiophron* can be identified with the following combination of characters: 1st discal cell of the forewing is more setose than basal and subbasal cells (often glabrous) in *Leiophron* (Fig. 3B)*,* but if not, then the ventral side of the 1st metasomal tergite (petiole) is not fused (Fig. 4C).

The exact age of the divergence between *Peristenus* and *Leiophron* is unknown, as the only known fossil record of Euphorini is a single specimen described as *Euphorus indurescens* Brues, found in Florissant, Colorado and dating back to Eocene at around 33.7–37 mya (Brues, 1910). In addition, both *Leiophron* and *Peristenus* have received little taxonomic attention outside of Europe, North America, and Asia (Loan, 1974a; Loan, 1974b; Chen & Van Achterberg, 1997; Belokobylskij, 2000b; Goulet & Mason, 2006; Stigenberg & Van Achterberg, 2016). We have included many undescribed species from Central and South America, Africa, and Papua New Guinea, which is unsurprising given the tremendous diversity of their major host Miridae. The first and second author are currently working on describing species from Papua New Guinea (J Stigenberg & YM Zhang, 2018, unpublished data), but a revision of the world Euphorini is needed.

### Validity of the genus *Mama*

The validity of the enigmatic genus *Mama*, described based on a single species *M. mariae* from eastern Russia (Belokobylskij, 2000a), has been questioned before by Simbolotti, Villemant & Van Achterberg (2004). Both *M. mariae* and *L. reclinator* have long, compressed, and spiny scapes (Fig. 4A), but due to the poor condition of the *L. reclinator* lectotype no definitive conclusion was made (Simbolotti, Villemant & Van Achterberg, 2004). With the consistent placement of the two species as sister taxa with short branch lengths in all three genes (Figs. S1–S3) and concatenated dataset (Fig. 2), and identical in morphology (the second author has examined the holotypes of *M. mariae* and *L. reclinator*), we synonymize *M. mariae* **syn. n**. as a junior synonym of *L. reclinator*, thus effectively dissolving the monotypic genus *Mama* **syn. n.** as a junior synonym of *Leiophron*. The distribution of *L. reclinator* likely spans across Eurasia, as specimens are found from eastern Russia to Sweden and the United Kingdom (Stigenberg & Van Achterberg, 2016).

### Updated generic concepts of Euphorini

**Tribe Euphorini Foerster 1862**

*Diagnosis.* Maxillary palp with five segments; labial palp with two to three segments; eye bare; first metasomal tergite petiolate; ovipositor slender and short, hardly protruding beyond metasoma; tarsal claws simple; forewing with marginal cell almost always equal or smaller than stigma (Fig. 3A); vein 3RSb (if present) strongly bent; vein r short or absent; vein 2M desclerotized; vein (RS+M)b absent; length of vein m-cu (if present) shorter than length of vein 2RS (Figs. 3A–3B).

**Genus** ***Peristenus*** **Foerster 1862**

***Peristenus*** **Foerster, 1862: 25; Shenefelt, 1969: 36 (as synonym of** ***Leiophron*** **Nees, 1818); (Shaw, 1985): 332. Type species (by original designation):** ***Microctonus barbiger*** **Wesmael, 1835 [=** ***Leiophron pallipes*** **Curtis, 1833].**

*Diagnosis.* Antennal segments 16–33; labial palp with three segments; occipital carina complete or interrupted dorsally; notaulus well-defined, crenulate, posteriorly joining just before posterior margin of mesoscutum; forewing with marginal cell large, complete; basal, subbasal, and 1st discal cells of forewing similarly setose (Fig. 3A); veins (RS+M)a, 1m-cu, 2CUa, 2CUb of forewing fully developed (Fig. 3A); veins rs-m, 2-1A of forewing absent; vein M+CU of forewing unsclerotized; veins 1cu-a and 1-1A of hindwing fully present; first metasomal tergite widened apically, ventrally fused or touching basally (Fig. 4B); metasomal tergites behind first tergite smooth; second suture absent; second tergite with lateral fold; hypopygium medium-sized, densely setose; ovipositor sheath slender, short, and densely setose; ovipositor slender, distinctly curved downwards.

*Biology.* Koinobiont endoparasitoids of Miridae (Hemiptera). The early instar nymphs are parasitized and the mature parasite larva emerge from either the mature host nymphs or the adults.

*Distribution.* Cosmopolitan except for Antarctic, limited to Papua New Guinea in Australasia.

**Genus** ***Leiophron*** **Nees, 1818**

***Leiophron*** **Nees, 1818: 303; Shenefelt, 1969: 35; Shaw, 1985: 326. Type species (designated by Viereck, 1914):** ***Leiophron apicalis*** **Haliday, 1833.**

***Euphoriana*** **Gahan, 1913: 433; Shenefelt, 1969: 33; Shaw, 1985: 326. Type species (by original designation):** ***Euphoriana uniformis*** **Gahan, 1913. Syn. by Loan, 1974b.**

***Euphoriella*** **Ashmead, 1900: 116; Shenefelt, 1969: 34; Shaw, 1985: 323. Type species (by monotypy & original designation):** ***Labeo incertus*** **Ashmead, 1887. Syn. By Chen & Van Achterberg, 1997**

***Euphorus*** **Nees, 1834: 360; Shenefelt, 1969: 35; Shaw, 1985: 326*):*** **Type species (by monotypy):** ***Euphorus pallicornis*** **Nees, 1834. Syn. by Muesebeck, 1958.**

***Mama*** **Belokobylskij, 2000:** **256; Stigenberg, Boring & Ronquist, 2015: 590. Type species (by monotypy & original desgination):** ***Mama mariae*** **Belokobylskij, 2000. Syn.n.**

*Diagnosis.* Antennal segments 14–20; labial palp with two to three segments; occipital carina usually widely interrupted dorsally; notaulus usually absent; marginal cell of forewing small, incomplete, or absent; 1st discal cell of forewing often more setose than basal and subbasal cells (Fig. 3B); forewing vein 3SRb ending far beforewing apex; forewing vein (RS+M)a present or absent; forewing vein 2M present; forewing vein M+CU largely unsclerotized; forewing vein 1M usually thickened; forewing vein 1CUb sclerotized or unsclerotized; forewing veins 2CUa and 2CUb absent; hindwing vein cu-a partly present or absent*;* first metasomal tergite nearly parallel-sided or slightly widened apically, ventrally variable: largely open, separated by a split at the midline (Fig. 3C), largely touching, or entirely fused (Fig. 3D); second and third tergites without lateral fold and second metasomal suture absent; hypopygium small, straight ventrally and setose; ovipositor hardly visible, usually shorter than 0.25 times first tergite, slender and curved downwards.

*Biology.* Koinobiont endoparasitoids of nymphal Hemiptera (Miridae and Lygaeidae) and Psocodea (Psocidae). The early instar nymph of the host is parasitized and the mature larva emerges from the mature host nymph or adult.

*Distribution.* Cosmopolitan except for Antarctic, limited to Papua New Guinea in Australasia.

## Conclusions

Using a multilocus phylogenetics approach and the most comprehensive taxon sampling of Euphorini to date, we were able to clarify the long standing taxonomic confusion within this tribe of economically important braconid wasps. The taxonomic uncertainty that has long impacted biological control studies of Euphorini is readily resolved with the revised generic concepts presented here, which reflects the strongly supported phylogenetic analyses, therefore providing clear distinguishing characters for the two genera *Peristenus* and *Leiophron*. With a phylogenetic framework to build upon, the next step should focus on the world revision of tribe Euphorini, with a strong alpha taxonomic component, as many of the species used in this study were undescribed or have an unknown biology.

##  Supplemental Information

10.7717/peerj.4783/supp-1 Table S1 Detailed collection locality information for all specimens used in this study

10.7717/peerj.4783/supp-2 Figure S1 *COI* gene tree for MrBayes *Peristenus* is colored red, and *Leiophron* is colored in blue, with subgenera within *Leiophron* shown in different colors (*Leiophron sensu stricto* in blue, *Euphorus* in purple, *Euphoriana* in green, *Euphoriella* in orange, and *Mama* in brown). Asterisks indicate strong nodal support ( ≥0.98 posterior probability).

10.7717/peerj.4783/supp-3 Figure S2 *28S* gene tree for MrBayes *Peristenus* is colored red, and *Leiophron* is colored in blue, with subgenera within *Leiophron* shown in different colors (*Leiophron sensu stricto* in blue, *Euphorus* in purple, *Euphoriana* in green, *Euphoriella* in orange, and *Mama* in brown). Asterisks indicate strong nodal support ( ≥0.98 posterior probability).

10.7717/peerj.4783/supp-4 Figure S3 *CAD* gene tree for MrBayes *Peristenus* is colored red, and *Leiophron* is colored in blue, with subgenera within *Leiophron* shown in different colors (*Leiophron sensu stricto* in blue, *Euphorus* in purple, *Euphoriana* in green, *Euphoriella* in orange, and *Mama* in brown). Asterisks indicate strong nodal support ( ≥0.98 posterior probability).

10.7717/peerj.4783/supp-5 Figure S4 Concatenated gene trees for MrBayes *Peristenus* is colored red, and *Leiophron* is colored in blue, with subgenera within *Leiophron* shown in different colors (*Leiophron sensu stricto* in blue, *Euphorus* in purple, *Euphoriana* in green, *Euphoriella* in orange, and *Mama* in brown). Asterisks indicate strong nodal support ( ≥0.98 posterior probability).

10.7717/peerj.4783/supp-6 Figure S5 Concatenated gene trees for RAxML *Peristenus* is colored red, and *Leiophron* is colored in blue, with subgenera within *Leiophron* shown in different colors (*Leiophron sensu stricto* in blue, *Euphorus* in purple, *Euphoriana* in green, *Euphoriella* in orange, and *Mama* in brown). Asterisks indicate strong nodal support ( ≥90 for bootstrap support).

10.7717/peerj.4783/supp-7 Figure S6 Concatenated gene trees for IQ-Tree *Peristenus* is colored red, and *Leiophron* is colored in blue, with subgenera within *Leiophron* shown in different colors (*Leiophron sensu stricto* in blue, *Euphorus* in purple, *Euphoriana* in green, *Euphoriella* in orange, and *Mama* in brown). Asterisks indicate strong nodal support ( ≥90 for ultrafast bootstrap support).

10.7717/peerj.4783/supp-8 Supplemental Information 1 Raw nexus file COI

10.7717/peerj.4783/supp-9 Supplemental Information 2 Raw nexus file 28S

10.7717/peerj.4783/supp-10 Supplemental Information 3 Raw nexus file CAD

10.7717/peerj.4783/supp-11 Supplemental Information 4 Raw nexus file of all 3 genes

10.7717/peerj.4783/supp-12 Supplemental Information 5 Raw phylip file of all 3 genes

## Additional Information and Declarations

### DNA Deposition

The following information was supplied regarding the deposition of DNA sequences: GenBank accession numbers MG913702–MG913792 and MG926854–MG926917.

### Data Availability

The following information was supplied regarding data availability: Files have been uploaded to Figshare: https://figshare.com/articles/Concatenated_File/6160247.
