# Supplementary material for: Multilocus phylogeny of the parasitic wasps in the tribe Euphorini (Hymenoptera: Braconidae) with revised generic classifications
Source: PeerJ. 2018 May 21;6:e4783. doi: 10.7717/peerj.4783 (PMC5967370; doi:10.7717/peerj.4783)
Supplement: Table S1 [file peerj-06-4783-s001.docx]

**Supplementary Table 1.** Detailed collection locality information for all specimens used in this study.

| **Taxon Label** | **Locality Label (verbatim)** |
| --- | --- |
| 07_Yves_Leiophron_PNG | Papua-New-Guinea, Province Madang, Mount Wilhelm 200m (-5.739897, 145.3297), 31.X—01.XI.2012, leg Dilu, Ray, Novotny, Leponce, Plot 1, understorey; Malaise - MAL-MW0200A-07/16-d07 |
| 08_Yves_Leiophron_PNG | Papua-New-Guinea, Province Madang, Mount Wilhelm 1700m (-5.759269, 145.2356), 27—28.X.2012, leg Valeba, Tulei, Novotny, Leponce, Plot 4, understorey; Malaise - MAL-MW1700A-03/16-d03 |
| 10_Yves_Leiophron_PNG | Papua-New-Guinea, Province Madang, Mount Wilhelm 2700m (-5.814968, 145.1580), 18—19.X.2012, leg Kua, Yalang, Novotny, Leponce, Plot 4, understorey; Malaise - MAL-MW2700D-03/16-d03 |
| AB016_Peristenus_KS | USA, Kansas, Riley Co. Konza Prairie Biol. Sta. 4B 39°06.65'N 96°35.75'W MT 25—29.X.2001, Zolnerowich, Kula, Brown |
| AB020_Peristenus_KY | USA, Kentucky, Harrison Co Silverlake Farm, Savanna 38°19.553'N 84°21.428'W MT 3 Hickory Edge [HI#7] 28.IV—5.V.2004, Hym. Inst. |
| AB023_Peristenus_KS | USA, Kansas, Riley Co. Konza Prairie Biol. Sta. Kings 39°06.20'N 96°35.77'W MT [HI#87] 1—4.V.2001, Zolnerowich, Kula, Brown |
| Euph_001_Euphorus_pallidistigma_SWE | Sweden, Öland Gårdby, 56°35'05.0N 16°36'54.6E 5.VII.2014, C.Hansson |
| Euph_017_Peristenus_JAP | Japan, Hyôgo Pref, Kobe-shi, Tanigami. 15.V—1.VI.2011 Malaise trap field. 34.7589 N, 135.1722 E. Leg. J. Stigenberg, H. Vårdal |
| Euph_020_Peristenus_SWE | Sweden, Skåne, Billebjär, 55.41188 13.19200, 16.V.2014, C.Hansson |
| Euph_083_Peristenus_HUN | Hungary, Köszeg NW outskirts of town, 21—27.VI.2010, MT in garden. |
| Euph_162_Leiophron_apicalis_SWE | Sweden, Öland, Gamla Skogsby (Kalkstad) mixed decidious forest, 27.V—27.VI.2014 Malaise trap, M. & C. Jaschhof. Jaschhof catalog #63/2014 |
| JS01000238_Leiophron_fascipennis_SWE | Sweden, Sm, Nybro kommun, Bäckebo, Grytsjöns naturreservat Old moisty haymaking meadow in forest edge N6311678 E1517066 (=TrapID 1001), 02.VII—12.VII.2005 (=coll. event ID 1332) |
| JS01000242_Leiophron_SWE | Sweden, Öl, Mörbylånga kommun, Frösslunda alvar, north eastern part, alvar pasture. N56°32.847´ E16°34.635´ (=TrapID 20) 05.VII—02.VIII.2005 (=coll.event ID 1498) |
| JS01000267_Leiophron_FRGU | French Guiana, Montagne des Chevaux, 15.VII—8.VIII.2011 |
| JS01000499_Mama_mariae_RUS | Russia, Primorskiy kray 10 km SE Partizansk, Novitskoe, forest, glades. 3-4.VIII.2010 Belokobylskij |
| JS01000515_Euphoriana_dispar_RUS | Russia, Primorskiy kray 10 km E Spassk-Dal'niy, 30.VI.2010 forest, glades. S. Belokobylskij |
| JS01000538_Euphorus_duploclaviventris_SWE | Sweden, Sm, Gränna kommun, Lönnemålen. Next to old cellar in Norway spruce forest w. big harvested ashes. N58°02.935´ E14°34.382´ (=Trap ID 17) 31.V—15.VI.2005 (=coll. event ID 1514) |
| JS01000539_Euphorus_oblitus_SWE | Sweden, Bl, Ronneby kommun, Tromtö, Tromtö nabb. Beech and oak forest. N56°08.944´ E15°28.801´ (=TrapID 23) 20.V—03.VI.2004 (=coll. event ID 449) |
| JS01000540_ Leiophron _deficiens_SWE | Sweden, Sk. Malmö. Limhamns Kalkbrott, Malaise trap 2-"planen", 26.VI—8.VII. 2009, leg. B.W.Svensson & Co |
| JS01000542_Leiophron_reclinator_SWE | Sweden, Sk. Malmö. Limhamns Kalkbrott, Malaise trap 1-"grafitti", 26.VI—8.VII. 2009, leg. B.W.Svensson & Co |
| JS01000547_ Leiophron _MAD | Madagascar, Finanrantsoa 16.XII.2011—1.I.2012 |
| JS01000552_Peristenus_SWE | Sweden, Sdm, Trosa kommun, Askö naturreservat. Malaise Trap N58°48,420", E17°40.437'. -1 moh. 14.VI—29.VI.2011. Loc.030-02 Leg.B.E. Bengtsson |
| JS01000553_Euphorus_basalis_SWE | Sweden, Sdm, Trosa kommun, Askö naturreservat. Malaise Trap N58°48,420", E17°40.437'. -1 moh. 14.VI—29.VI.2011. Loc.030-02 Leg.B.E. Bengtsson |
| JS01000554_Euphorus_fulvipes_SWE | Sweden, Sdm, Trosa kommun, Askö naturreservat. Malaise Trap N58°48,420", E17°40.437'. -1 moh. 14.VI—29.VI.2011. Loc.030-02 Leg.B.E. Bengtsson |
| JS068_Leiophron_COL | Colombia, Boyaca, Iguaque M. 3533 |
| JS120_Leiophron_THA | Thailand, Trang Pr., 140m. 20—27.I.2005 |
| JS129_Leiophron_THA | Thailand, Trang Pr., 140m. 20—27.I.2005 |
| PNG_5_Leiophron | Papua-New-Guinea, Province Madang, Mount Wilhelm 3200m (-5.806944, 145.0721), 20—21.X.2012, leg Dahl, Kaupa, Novotny, Leponce, Plot 3, understorey; Malaise - MAL-MW3200C-05/16-d05; P3374 |
| PNG_6_Leiophron | Papua-New-Guinea, Province Madang, Wanang 3 station (-5.22767, 145.0797) 175m, 18—19.XI,2012, leg Basset, Plot 3, understorey; Malaise - MAL-WAN03-D01 P4932 |
| PNG_7_Leiophron | Papua-New-Guinea, Province Madang, Wanang 3 station (-5.22767, 145.0797) 175m, 02—03.XII.2012, leg Basset, Plot 1, understorey; Malaise - MAL-WAN01-D15 P4914 |
| YMZ038_Peristenus_GER | Germany, Schleswig-Holstein, 11.VIII.2001 |
| YMZ077_Leiophron_uniformis_MB | Canada, Manitoba, Carman, 17.VI.2013, Y, Miles Zhang |
| YMZ081_Euphoriella_MB | Canada, Manitoba, Carman, 17.VI.2013, Y, Miles Zhang |
| YMZ124_Peristenus_mellipes_MB | Canada, Manitoba, Beaudry Provincial Park, 10.X.2014 |
| YMZ132_Leiophron_KY | USA, Kentucky, Franklin Co. Cove Springs Park 38 13.237N 84 51.414W MT2 wooded clearing [HI#235] 14—21.VI.2005 K. Pitz |
| YMZ133_Leiophron_KY | USA, Kentucky, Franklin Co. Lexington: Tee It Up Golf 37 58'39"N 84 24'59"W MT: malaise trap [HI#200] 10—17.IX.2004, B. Sharanowski |
| YMZ134_Leiophron_WV | USA, West Virginia, Hardy Co. 3mi NE Mathias 38 55'N 78 49'W 28.V—4.VII.2004, MT, David R. Smith |
| YMZ136_Leiophron_KY | USA, Kentucky, Fayette Co. Lexingon 1118 Slashes Rd 83 29'07"w 38 01'45"N MT: M. Sharkey X.2003 |
| YMZ139_Leiophron_uniformis_FRA | France, Herault Baillarguet CSIRO lab 43 41'12"N 3 52'24'E 3—15.V.1993 P.G. Mason champ sauvage, MT |
| YMZ141_Leiophron_THA | Thailand, Chiang Rai Prov Doi Luang National Park Namtok Pu Kaeng, 540m 19 26'N 99 42'E MT: [HI#62] 16.III.2002 Coll: Mercury Vapour Lamp |
| YMZ142_Peristenus_MAD | Madagascar, Fianarantsoa Prov Parc Nat, Ranomafana, radio tower@ forest edge 22—26 XI.2001, ele 1130m Calif. Aca. Sci. 21 15.05'S 47 24. 43'E MA-02-09B-04 Caslot 014023, MT, mixed tropical forest Coll: R. Harin' Hala |
| YMZ145_Leiophron_COL | Colombia, Cundinamarca PNN Sumapaz Bocatoma. Cerro el zapato 4 14'N 74 12'W 3560m MT 18.XI—4.XII.2002 A. Patino Leg,M.3443 |
| YMZ146_Leiophron_COL | Colombia, Cundinamarca PNN Sumapaz Bocatoma. Cerro el zapato 4 14'N 74 12'W 3560m MT 18.XI—4.XII.2002 A. Patino Leg,M.3443 |
| YMZ148_Euphoriella_GUA | Guatemala, Suchitepeque Finca Moca Grande hill behindlake, MT 23—24.II.1995, D. Quintero A. |
| YMZ211_Peristenus_dayi_MB | Canada, Manitoba, Nopiming Provincial Park, 06/2012, Sharanowski Lab |
| YMZ335_Peristenus_howardi_AB | Canada, Lethbridge, Peenaquim Park, 14.VII.2011 |
| YMZ341_Peristenus_relictus_LAB_COLONY | USA, New Jersey, Philip Alampi Beneficial Insect Lab, New Jersey Department of Agriculture, 09.VI.2016 |
| YMZ343_Peristenus_digoneutis_LAB_COLONY | USA, New Jersey, Philip Alampi Beneficial Insect Lab, New Jersey Department of Agriculture, 09.VI.2016 |
| YMZ345_Leiophron_KY | USA, Kentucky, Herndon Farm 38 33 25N 084 59 35W MT 6 Shed, f-c interface 5.VIII—17.VIII 2009, 151m Hym Institute |
| YMZ346_Peristenus_WI | USA, Wisconsin, La Crosse Co. nr West Salem 43 54 22.22N 91 10 52 W 21—30.V.2010, alt 387 MT A.M. Shorter |
| YMZ349_Peristenus_IL | USA, Illinois, Lee Co, Richardson Wildlife Foundation 41 32 26.91N 89 11 12.79W 20—29.VII.2010. 252m Terry Moyer |
| YMZ351_Leiophron_VA | USA, Virginia, Hanover Co. 2.39k NW Vontay N 37.765172 W 77.775934 MT 28.V—11.VI.2011, AV Evans, JC Ludwing |
| YMZ356_Peristenus_WI | USA, Wisconsin, La Crosse Co. nr West Salem 43 54 22.22N 91 10 52 W 11—21.VI.2010. alt 387 MT A.M. Shorter |
| YMZ358_Euphoriella_KY | USA, Kentucky, Franklin Co. Cove Springs Park 38°13.178'N 84°51.325W MT 1: Floodplain [HI#234] 14—21.VI.2005. K. Pitz |
| YMZ359_Euphoriella_FL | USA, Florida, Alachua Co. Gainesville, AEI 29°35'53.6"N 82°21'54.8"W IV.2005, MT D.B. Wahl |
| YMZ361_Leiophron_AZ | USA, Arizona, Cochise Co. Bishee, 1429 Franklin Street, Malise in dry wash, 1585m 31.4038°N 109.9262°W 18—28.V.2015 AS Menke |
| YMZ363_Euphoriella_COL | Colombia, Valle del Cauca PNN Farallones de Cali Cgto. La Meseta 3°34'N 76°40'W 2080m Malaise 10—25.II.2004 S. Sarria & M. Losso Leg. 4555 |
| YMZ364_ Leiophron _CR | Costa Rica, Prov. Heredia 6km ENE Vara Blanca 10°11'N 84°07'W 2000m 20/M/18/038, 10.III.2002 INBio-OET-transect |
| YMZ365_Euphoriella_CR | Costa Rica, Prov. Heredia 6km ENE Vara Blanca 10°11'N 84°07'W 2000m 20/M/12/072, 9.IV.2002 INBio-OET-ALAS-transect |
| YMZ366_Euphoriella_GUA | Guatemala, Peten Parq. Nac. Tikal 17.24030 -89.62207 6m-270m 22.V.2009. LLAMA#Wa-B-05-2-01 |
| YMZ367_Leiophron_HON | Honduras, Atlantida 7km SSW Tela 15.72417 -87.45187 150m-190m 15.VI.2010, LLAMA#Wa-C-08-2-all |
| YMZ368_Leiophron_VEN | Venezuela, Aragua Rancho Grande 1140m 1—6.III.1995. R.W.Brooks. FIT |
| YMZ370_Euphoriella_PER | Peru, Wayqecha Oso S13°11.370 W71°35.074 16—28.VII.2014 Sharanowski Lab |
| YMZ371_Leiophron_PER | Peru, Wayqecha Oso S13°11.370 W71°35.074 16—28.VII.2014 Sharanowski Lab |
| YMZ372_Peristenus_PER | Peru, Wayqecha Oso S13°11.370 W71°35.074 16—28.VII.2014 Sharanowski Lab |
| YMZ373_Leiophron_THA | Thailand, Petchaburi Kaeng Krachan NP Pa La-U/Haui Palao Forest Unit 3 12°32.149'N 99°28.265'E Malaise Trap 18—25.I.2009 Thongbai leg. T4566 |
| YMZ375_Leiophron_KEN | Kenya, Eastern Prov. Njuki-ini Forest, nr. Forest station, 1455m 0.51660o S, 37.41843o E 15—29.IX.2008 MT R. Copeland |
| YMZ376_Leiophron_THA | Thailand, Trang Pr. Khoa Chang, Forest Research Stn. 7°33'2"N 99°47'23"E 75m 21—26.I.2005 D. Lohman |
| YMZ377_Leiophron_THA | Thailand, Trang Pr. Khoa Chang, Forest Research Stn. 7°33'2"N 99°47'23"E 75m VIII.2005 D. Lohman |
| YMZ378_Leiophron_THA | Thailand, Trang Pr. Khoa Chang, Forest Research Stn. 7°33'2"N 99°47'23"E 75m VII.2005 D. Lohman |
| YMZ380_Leiophron_CON | Congo, Dept Pool Iboubikro, Lesio-Looun Pk, 330m 03°16.196S, 015°28.267E MT 26.XI—7.XII.2008 Sharkey+Braet A131 |
| YMZ382_Leiophron_KOR | South Korea, Ganwondo Chuncheon, Man-myeon Balsan, 300m, MT in forest 37°43.29'N 127°37.73'E 30.IX—11.XI.2006 Tripotin rec. |
| YMZ383_Leiophron_KOR | South Korea, Chungnam Daejon-si Wadong 36°24.02'N 127°25.98E 19.VI.—16.VII.2006 P. Tripotin, MT, Forest edge, wild rose patch |
| YMZ384_Leiophron_KOR | South Korea, Ganwondo Pyeonchang, Yongpyeong - myeon Nodong Valley, 900m 37°42.08'N 128°28.89'E 31.V.—5.VI.2006 P. Tripotin, MT in forest |
| YMZ385_Leiophron_KOR | South Korea, Ganwondo Pyeonchang, Yongpyeong - myeon Nodong Valley, 900m 37°42.08'N 128°28.89'E 31.V.—5.VI.2006 P. Tripotin, MT in forest |
| YMZ386_Leiophron_CON | Congo, Dept Pool Iboubikro, Lesio-Looun Pk, 330m 03°16.196S, 015°28.267E MT4 20.X.2008 Sharkey+Braet A134 |
| YMZ388_Leiophron_pallidistigma_KOR | South Korea, Chungnam Daejon-si Wadong 36°24.02'N 127°25.98E 28.V.—19.VI.2006 P. Tripotin, MT, Forest edge, wild rose patch |
| AB102 Microctonus (Perilitini) | USA, Kentucky, Lexington, Tee It Up Golf 37°58'39"N 84°24'59"W MT [HI#200] 10—17.IX.2004, B. Sharanowski |
| JS01000218 Townesilitus (Townesilitini) | Sweden, Sk, Ystads kommun, Sandhammaren strand, Järahusen.Border between forest and sandhill dunes. N61°42.074´ E13°98.890´ (= TrapID 1005) 22.v - 15.vii.2005 (=coll. event ID 1419) |
| JS115 Chrysopophthorus (Helorimorphini) | Colombia, Magdelena, PNN Tayrona Canaveral (30m). 3-22.xi.2000 |
