# Supplementary figures and images for: Multilocus phylogeny of the parasitic wasps in the tribe Euphorini (Hymenoptera: Braconidae) with revised generic classifications
Source: PeerJ. 2018 May 21;6:e4783. doi: 10.7717/peerj.4783 (PMC5967370; doi:10.7717/peerj.4783)

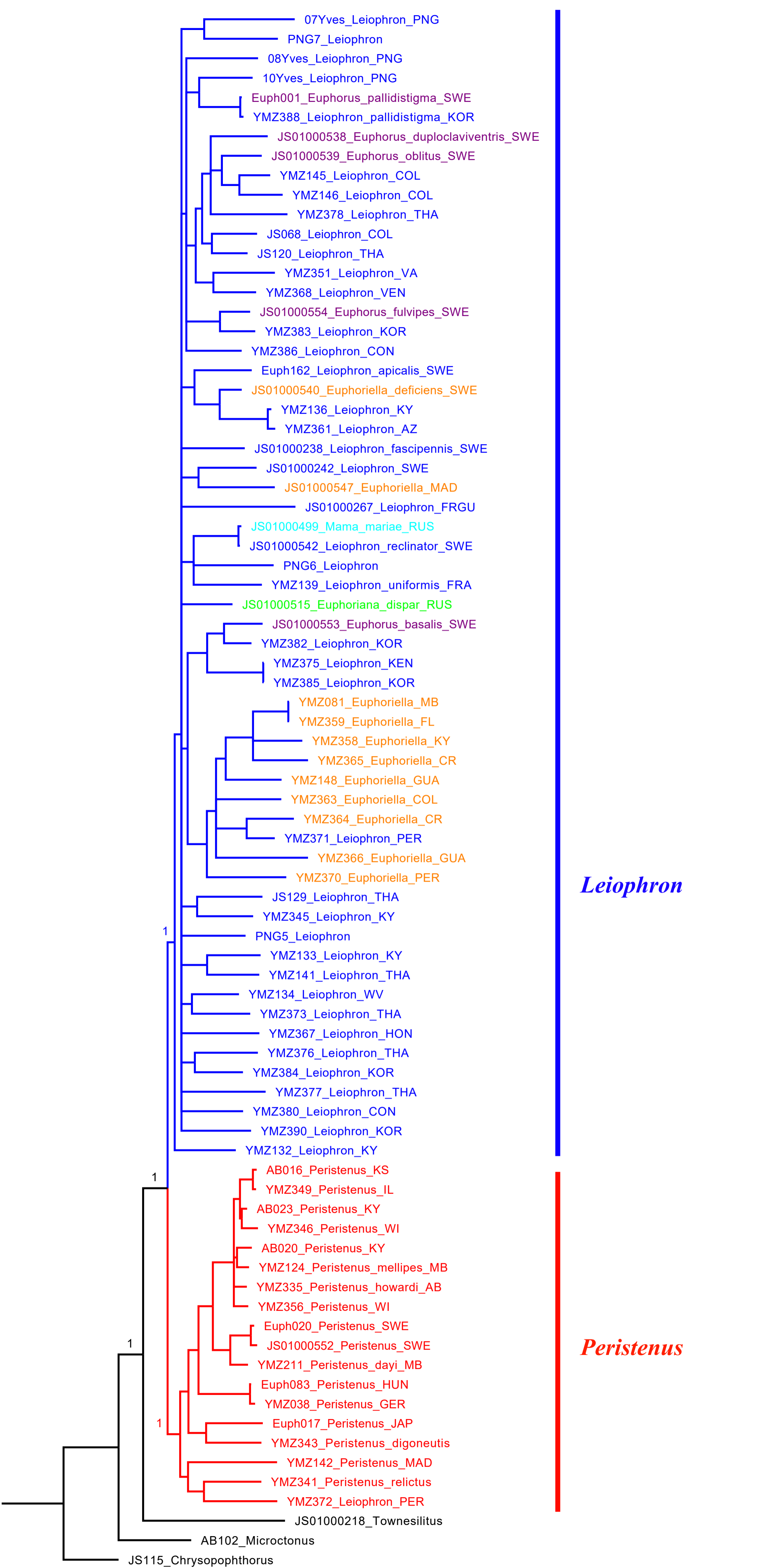

Supplement: Figure S1 — Peristenus is colored red, and Leiophron is colored in blue, with subgenera within Leiophron shown in different colors (Leiophron sensu stricto in blue, Euphorus in purple, Euphoriana in green, Euphoriella in orange, and Mama in brown). Asterisks indicate strong nodal support ( ≥0.98 posterior probability). [file peerj-06-4783-s002.pdf]

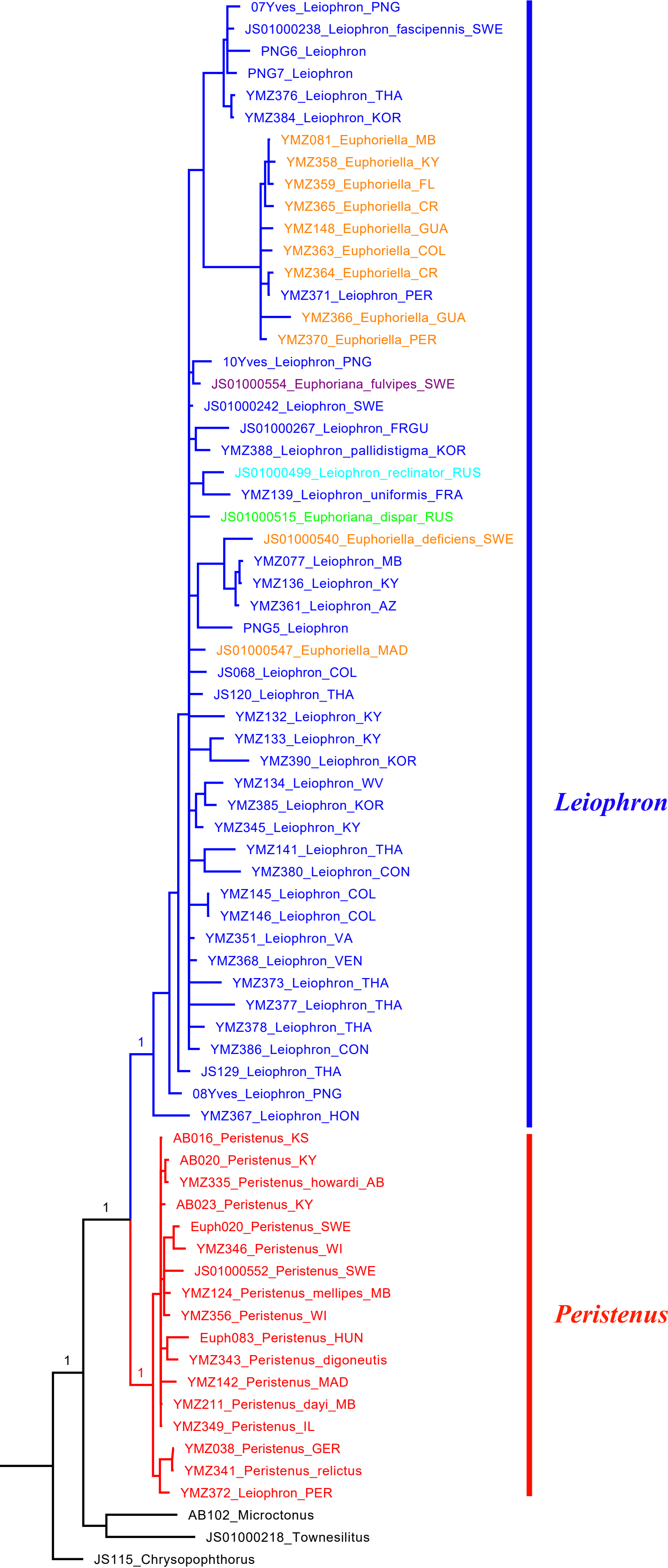

*Leiophron*

*Peristenus*

Supplement: Figure S2 — Peristenus is colored red, and Leiophron is colored in blue, with subgenera within Leiophron shown in different colors (Leiophron sensu stricto in blue, Euphorus in purple, Euphoriana in green, Euphoriella in orange, and Mama in brown). Asterisks indicate strong nodal support ( ≥0.98 posterior probability). [file peerj-06-4783-s003.pdf]

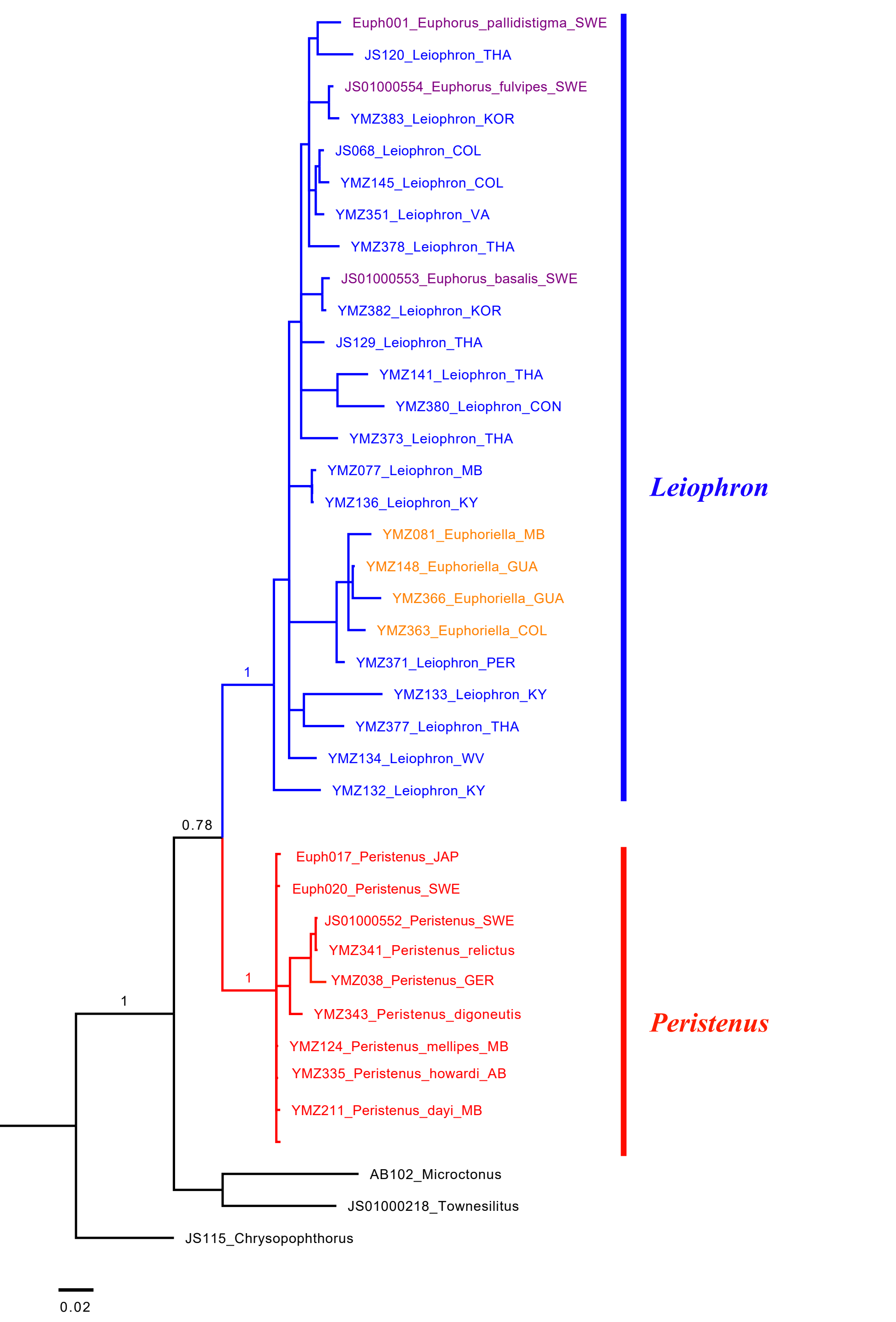

Supplement: Figure S3 — Peristenus is colored red, and Leiophron is colored in blue, with subgenera within Leiophron shown in different colors (Leiophron sensu stricto in blue, Euphorus in purple, Euphoriana in green, Euphoriella in orange, and Mama in brown). Asterisks indicate strong nodal support ( ≥0.98 posterior probability). [file peerj-06-4783-s004.pdf]

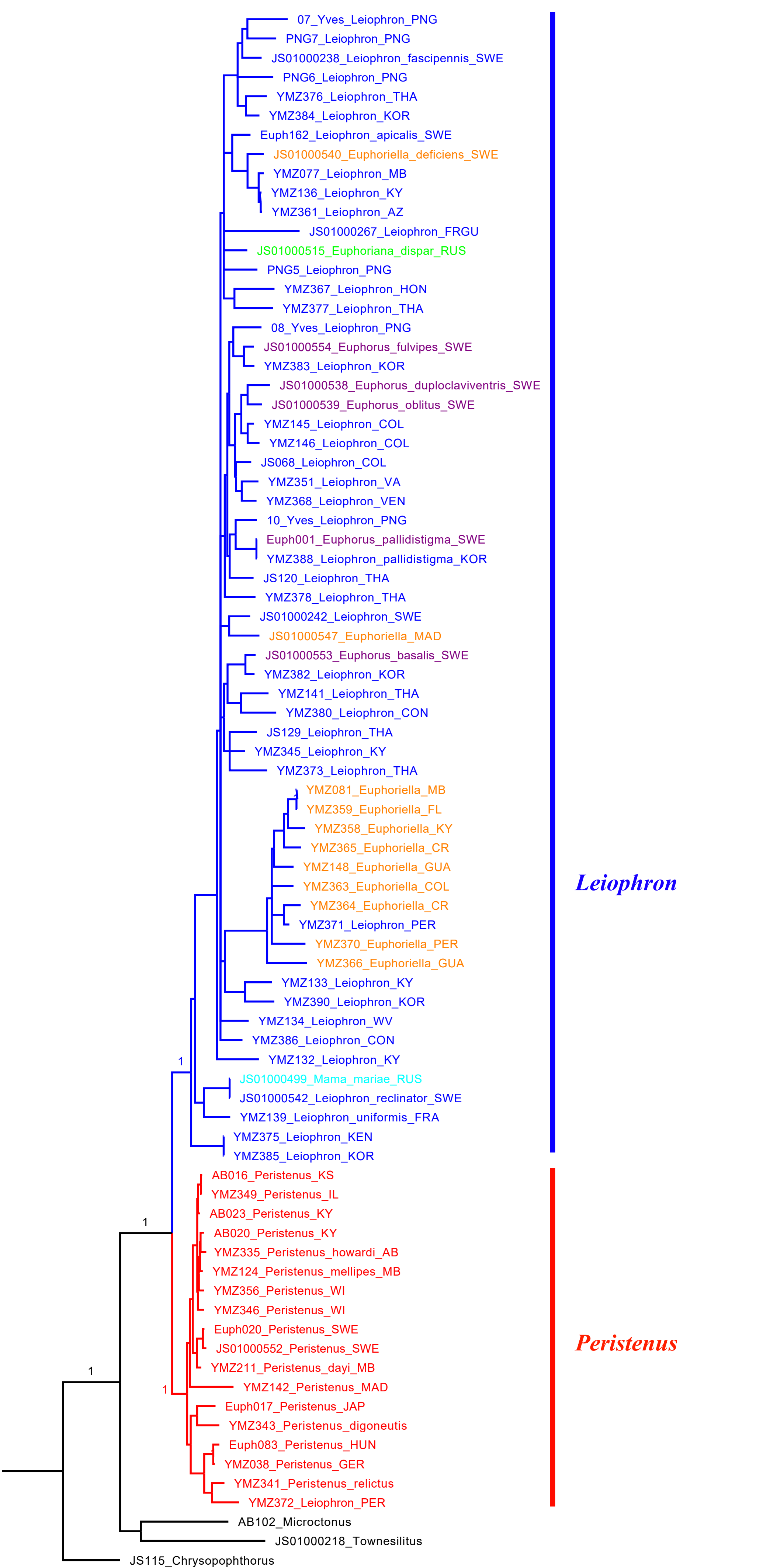

Supplement: Figure S4 — Peristenus is colored red, and Leiophron is colored in blue, with subgenera within Leiophron shown in different colors (Leiophron sensu stricto in blue, Euphorus in purple, Euphoriana in green, Euphoriella in orange, and Mama in brown). Asterisks indicate strong nodal support ( ≥0.98 posterior probability). [file peerj-06-4783-s005.pdf]

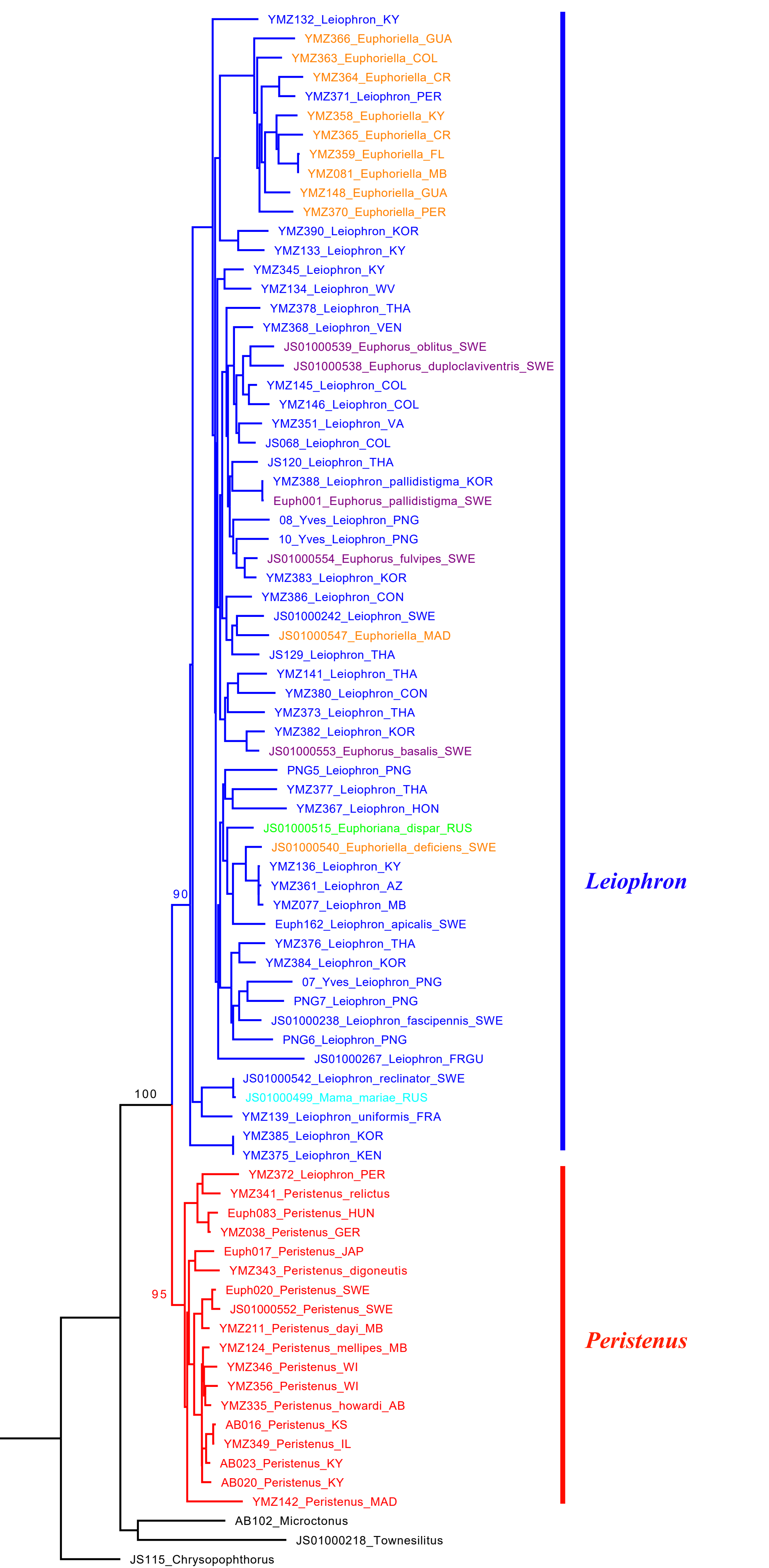

*Leiophron*

*Peristenus*

Supplement: Figure S5 — Peristenus is colored red, and Leiophron is colored in blue, with subgenera within Leiophron shown in different colors (Leiophron sensu stricto in blue, Euphorus in purple, Euphoriana in green, Euphoriella in orange, and Mama in brown). Asterisks indicate strong nodal support ( ≥90 for bootstrap support). [file peerj-06-4783-s006.pdf]

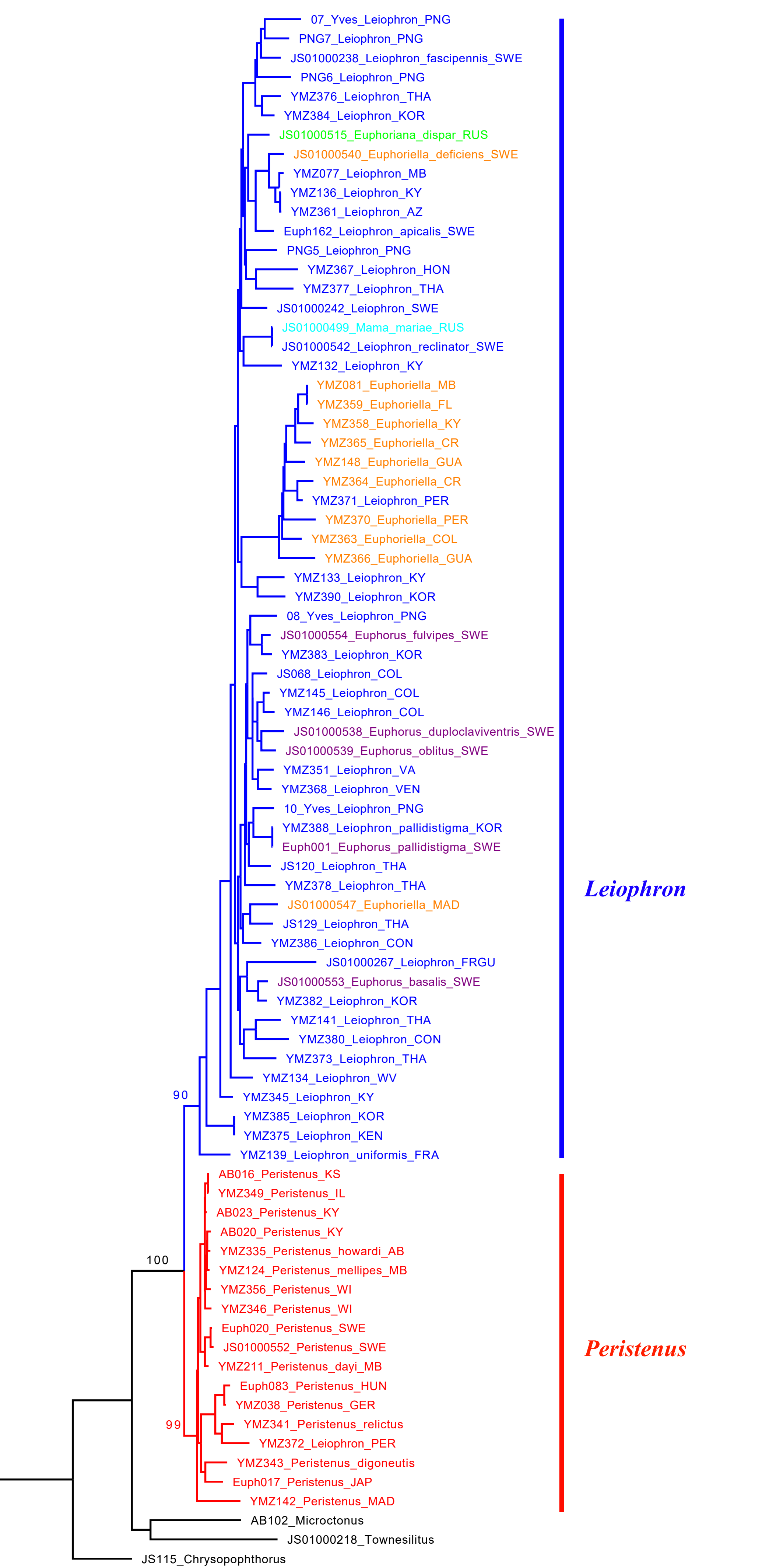

*Leiothorax*

*Peristhenus*

Supplement: Figure S6 — Peristenus is colored red, and Leiophron is colored in blue, with subgenera within Leiophron shown in different colors (Leiophron sensu stricto in blue, Euphorus in purple, Euphoriana in green, Euphoriella in orange, and Mama in brown). Asterisks indicate strong nodal support ( ≥90 for ultrafast bootstrap support). [file peerj-06-4783-s007.pdf]
